# Comprehensive Explorations and Preliminary Experimental Verification of RNA Modification-Related Diagnostic Markers in the Subtype Classification of Peripheral Blood-Derived Mononuclear Cells Derived from Post-Traumatic Stress Disorder Patients

**DOI:** 10.3390/diseases13100323

**Published:** 2025-10-01

**Authors:** Lesheng Wang, Gaomeng Luo, Sha Liu, Zhipeng Xu, Wei Wei, Xiang Li

**Affiliations:** 1 Department of Neurosurgery, Zhongnan Hospital of Wuhan University, Wuhan 430071, China; wanglesheng@whu.edu.cn (L.W.); luogaomeng@whu.edu.cn (G.L.); 2 Brain Research Center, Zhongnan Hospital of Wuhan University, Wuhan 430071, China; 3 Department of General Practice, Zhongnan Hospital of Wuhan University, Wuhan 430071, China; sha.liu@whu.edu.cn; 4 Department of Neuropsychology, Zhongnan Hospital of Wuhan University, Wuhan 430071, China; xzhp14@sina.com; 5 Medical Research Institute, Wuhan University, Wuhan 430071, China; 6 Frontier Science Center for Immunology and Metabolism, Wuhan University, Wuhan 430071, China; 7 Sino-Italian Ascula Brain Science Joint Laboratory, Zhongnan Hospital of Wuhan University, Wuhan 430071, China

**Keywords:** post-traumatic disorder, RNA modification, genes, regulator, N6-methyladenosine, 5-methylcytosine, N1-methyladenosine, N7-methylguanosine, pseudouridine

## Abstract

Background: The precise role of RNA modification in post-traumatic stress disorder (PTSD) remains incompletely understood. This study aims to elucidate the effects of five common RNA modifications in PTSD, specifically m^6^A, m^5^C, m^1^A, m^7^G, and ψ. Methods: We extracted data from the GEO repository to conduct a series of bioinformatics analyses. These included differential analysis to identify key regulators of five common RNA modifications, model construction using random forest (RF), least absolute shrinkage and selection operator (LASSO), and nomogram techniques, as well as consensus clustering of RNA modification subtypes. Furthermore, GO enrichment analysis was performed on DEGs associated with various RNA modification patterns. Immune cell infiltration was assessed using PCA and ssGSEA. RT-qPCR was performed to validate RNA modification-related genes (RMGs). Results: Twenty-one differentially expressed RMGs were identified. LASSO and RF intersection yielded eight signature genes (YTHDC1, IGFBP1, IGF2BP1, ALKBH5, NSUN4, TET2, TET3, WDR4) that robustly diagnosed PTSD (AUC = 0.804). Furthermore, these feature genes were validated using RT-qPCR, which was basically consistent with the results of bioinformatics analysis. Consensus clustering analysis may reveal two distinguishable subtypes: clusterA marked by high immunoinflammation, and clusterB characterized by high-neuroendocrine dysregulation. Conclusions: RMGs may play a crucial role in the pathogenesis of PTSD. Analyzing RNA modification patterns could offer potential diagnostic markers and help to guide immunotherapeutic approaches or neurotransmitter system interventions for PTSD in the future.

## 1. Introduction

Post-traumatic stress disorder (PTSD) is a mental health condition triggered by experiencing or witnessing a traumatic event, leading to the development of distressing recollections that impede the individual’s capacity to function effectively and maintain a typical lifestyle [1]. The prevalence of PTSD remains high globally, substantially ranging from 1 to 8% [2,3]. The main treatment modalities include pharmacological interventions, psychotherapy, and cognitive behavioral therapy [4,5]. However, these therapies have some disadvantages, including high costs, side effects, symptom relapse after cessation of treatment, and lifelong therapy. The effects of current pharmacologic or psychological treatment remain unsatisfactory. Therefore, it is imperative to promptly elucidate the specific molecular pathways implicated in PTSD.

Neurodegenerative diseases and mental disorders have been associated with changes in epigenetic modifications, encompassing alterations in DNA and RNA modifications, histone modifications, noncoding RNA modifications, and chromatin rearrangements [6,7,8]. Of these epigenetic modifications, RNA modifications refer to post-transcriptional chemical alterations that covalently add chemical groups or introduce structural changes without altering the underlying RNA sequence. They play critical roles in regulating diverse biological processes such as RNA stability, localization, splicing, translation, and degradation, thereby exerting broad influences on gene expression [9]. To date, more than 170 distinct types of RNA modifications have been identified [10]. Among them, N6-methyladenosine (m^6^A), 5-methylcytosine (m^5^C), N1-methyladenosine (m^1^A), N7-methylguanosine (m^7^G), and pseudouridine (ψ) are the common abundant forms of RNA modifications [11]. To date, RNA modifications are strongly associated with neurological disorders, metabolic diseases, viral infections, tumors, and other diseases. Present studies have focused on the crosstalk between RNA modifications and cervical cancers [12,13], lung cancers [14,15,16], breast cancers [17,18], and glioma [19,20,21]. While the role of RNA modifications in neuropsychiatric disorders is an emerging field, growing evidence indirectly suggests their involvement in stress-related pathophysiology. Specially, altered expression of m^6^A regulators has been associated with PTSD-like behaviors in animal models [22,23,24]. However, the comprehensive landscape of RNA modifications in patients with PTSD remains largely uncharted in contrast to PTSD animal models, necessitating further systematic interrogation to elucidate its specific role in disease etiology and maintenance.

**Figure 1:**
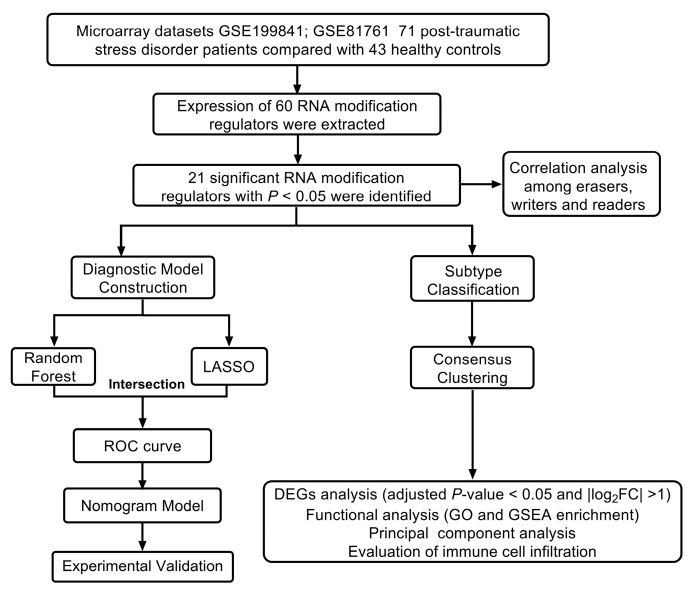
Flow chart of this study.

To address this issue, we conducted a comprehensive analysis of the impact of RNA modification-related genes (RMGs) that encode writers, erasers, readers, and other regulators involved in dynamically regulation of modified RNAs on the identification of diagnostic biomarkers and subtypes of PTSD using GEO database with samples from blood. A gene model for predicting susceptibility to PTSD was created using 8 feature RMGs to offer significant clinical advantages for patients. Additionally, RT-qPCR experiments confirmed the 8 feature RMGs, showing similar expression levels to the bioinformatics findings. The workflow diagram is depicted in Figure 1.

## 2. Materials and Methods

### 2.1. Data Acquisition and Preprocessing

Using the GEO (http://www.ncbi.nlm.nih.gov/geo/, accessed on 5 June 2024) database, we searched for expression microarrays matching terms (“Post-traumatic stress disorder”, “Gene expression”, “Microarray”) associated with PTSD. “Homo sapiens” was the filtering organism for the top, “Series” was the filtering entry type, and “Expression profiling by array” was the filtering study type. Datasets from GEO can be obtained as raw data and as series matrix files. Finally, four datasets that met the criteria were included in the analysis. Samples from GSE19984 and GSE81761 were used as the training set (Table 1), while GSE97356 and GSE77164 were used as external validation datasets.

Prior to normalization, robust multiarray analysis (RMA) was conducted, and probes were converted into gene symbols using the annotation information of the normalized data. In order to calculate the final expression value, multiple probes were used to measure the average expression value for each probe. Various datasets or the same dataset across different platforms were utilized to extract multiple datasets containing identical gene symbols. The removeBatchEffect function from the limma package in R software (version 4.2.1) was employed to eliminate batch effects by categorizing different datasets or platforms as distinct batches. Principal component analysis (PCA) plots were utilized to illustrate the impact of batching on samples. In order to distinguish between two distinct groups, each sample was labeled as either “PTSD” or “Con” to delineate samples indicative of post-traumatic stress disorder from those representing healthy controls.

### 2.2. Extraction and Differential Analysis of RMGs

The Limma R package (version 4.2.1.) was employed to detect and display differentially expressed RMGs between PTSD and Con samples with statistical value (*p* < 0.05). The ggpubr package was utilized for generating a boxplot, while the pheatmap package was utilized for generating a heatmap. A chromosomal map of each identified RMG was constructed using the Perl language and visualized using the RCircos R package.

### 2.3. Correlation Analysis of RMGs

For exploring and visualizing the correlations among these RMGs, correlation analysis was performed using R packages “corrplot”. In this study, the threshold for statistical significance was established at a *p*-value of 0.001 or higher.

### 2.4. Construction of the RF, LASSO, and Nomogram Models

In order to identify key RMGs and forecast the occurrence of PTSD, random forests (RFs) and least absolute shrinkage and selection operator (LASSO) were constructed. The random forest algorithm was used to evaluate feature importance. This algorithm constructed 500 classification trees and evaluated feature importance based on MeanDecreaseGini. LASSO regression was implemented using the “glmnet” R package, setting the response type to binary classification (binomial) and the regularization parameter α = 1. Receiver operating characteristics (ROCs) were generated to assess the performance of the models. Utilizing the “rms” package within the R programming environment, a nomogram model was developed to forecast the likelihood of PTSD occurrence. The comparison of predicted and observed values was depicted using a calibration plot, while a decision curve analysis (DCA) and a clinical impact curve were utilized to evaluate the efficacy and benefits of the model.

### 2.5. Consensus Clustering Analysis of Differentially Expressed Levels of Differentially Expressed Immune Infiltration

The R software was employed to implement an unsupervised clustering algorithm using the consensusClusterPlus package, leading to the categorization of PTSD samples into subtypes based on the degree of consensus in the feature RMGs. Delta area plots and consensus cumulative distribution function (CDF) curves were employed to ascertain the optimal number of subtypes. Additionally, PCA was utilized to evaluate the classification, while ssGSEA was employed to determine the immune cell abundance of PTSD patients. The immune cell abundance of PTSD patients was calculated utilizing ssGSEA. Furthermore, immuno-correlation analysis was performed based on the ssGSEA score, and the results were visually represented through the generation of heatmaps and boxplots.

### 2.6. Identification of RNA Modification Gene Subtypes Through Analysis of DEGs Within Various RNA Modification Subtypes

GO enrichment analysis was performed on DEGs across RNA modification subtypes. Gene set enrichment analysis and Kyoto Encyclopedia of Genes and Genomes (GSEA-KEGG) were utilized to compare the two RNA modification subtypes. Following this, DEGs were utilized in an unsupervised clustering algorithm to categorize samples of PTSD into subtypes based on RNA modification genes. Additionally, PCA method was used to calculate an RNA modification score. RNA modification scores were compared between different subtypes of RNA modifications and gene subtypes associated with RNA modifications. A Sankey diagram was generated utilizing the R packages “ggalluvial,” “ggplot2,” and “dplyr” to visually represent the connections and coherence between RNA modification subtypes, genes, and scores.

### 2.7. Differential Analysis of Genes Related to PTSD in Various Subtypes

Various genes associated with PTSD were selected from pertinent literature sources for comparative analysis within various RNA modification subtypes and gene subtypes, including but not limited to solute carrier family 6 member 4 (SLC6A4), ADCYAP receptor type I (ADCYAP1R1), solute carrier family 6 member 3 (SLC6A3), catechol-O-methyltransferase (COMT), FKBP prolyl isomerase 5 (FKBP5), dopamine receptor D2 (DRD2), corticotropin releasing hormone receptor 1 (CRHR1), steroid 5 alpha-reductase 2 (SRD5A2), solute carrier family 6 member 2 (SLC6A2), interleukin 6 (IL6), interleukin 1 beta (IL1B), protein kinase C alpha (PRKCA), tryptophan hydroxylase 2 (TPH2), and tumor necrosis factor (TNF) [25].

### 2.8. Experimental Validation by RT-qPCR

This study received approval from the Ethical Commission of Zhongnan Hospital of Wuhan University (ethical approval code: No. 2022174), and informed consent was obtained from all participants. A cohort consisting of eleven PTSD individuals and twelve healthy controls was assembled for the purpose of validating candidate genes through the collection of venous blood samples (Table 2). We manipulated human peripheral blood monocytes (HPBMs) as described in previous studies [26]. To extract total RNA from HPBMs, 500 nL of Vezol reagent (Vazyme Inc., Nanjing, China) was applied following the manufacturer’s instructions. Following retrotranslation of RNA, the primers amplifying RNA modification related genes were used to detect the relative expression of RMGs using 10 uL SYBR Green qPCR SuperMix (Vazyme Inc., Nanjing, China) on RotorGene Q (Qiagen, Shanghai, China). The relative quantitative analysis was conducted by 2^−ΔΔCT^ method, with each qPCR reaction being conducted in duplicate for every sample and replicated twice. Table 3 displays the gene-specific detection primers for 8 feature RMGs provided by Sangon Biotechnology Co., Ltd. (Shanghai, China).

### 2.9. Statistical Analysis

The Wilcoxon test was used to identify differentially expressed RMGs between PTSD and Con samples in the GEO database. The relationships among RMGs were assessed using linear regression analysis. DEGs between clusters were identified using an empirical Bayes moderated *t*-test. Statistical analysis was performed using unpaired two-tailed *t* tests with Welch’s correction applied in RT-qPCR data analysis, or Kruskal–Wallis tests applied in bioinformatics analysis, with statistical significance set at *p* < 0.05.

## 3. Results

### 3.1. Normalized the Databases Downloaded from GEO

**Figure 2:**
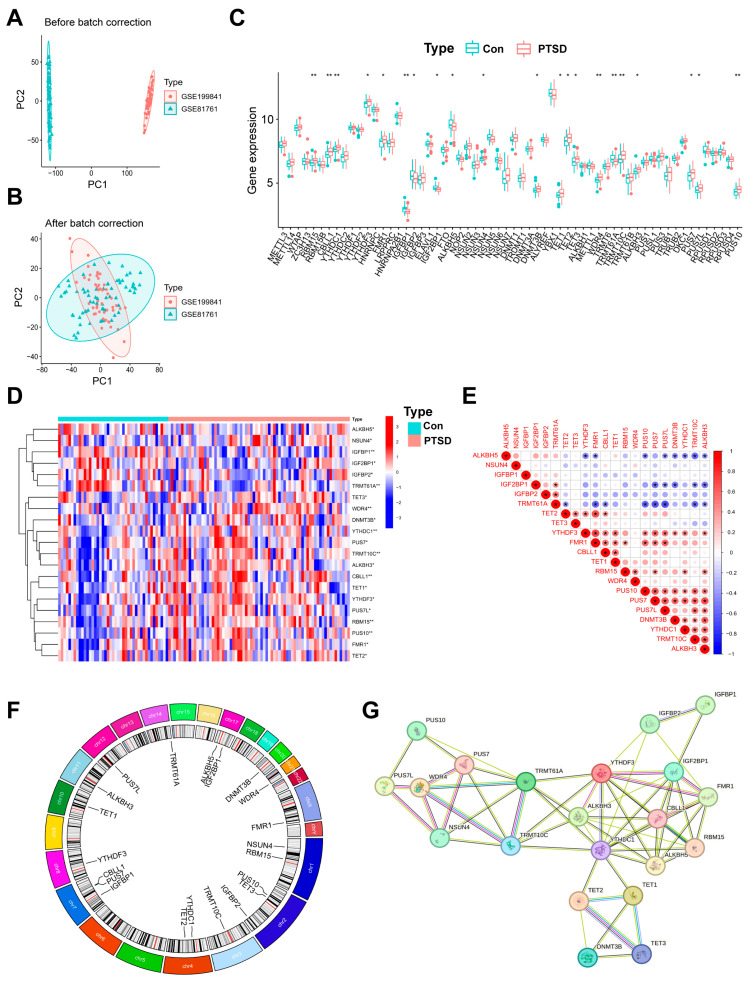
Identification of 60 RMGs in Con and PTSD samples. (**A**,**B**) A before-and-after de-batching process of 114 samples from the two databases. (**C**) A boxplot illustrating the differential expression of the 60 RMGs between PTSD and Con. (**D**) A heatmap showing 21 differential RMGs in the PTSD and Con. (**E**) Correlations among 21 RMGs. (**F**) The chromosomal positions of 21 RMGs. (**G**) A network analysis visualizing the interactions among RNA modification regulatory proteins using data from the String database. Statistical annotations: * *p* < 0.05, and ** *p* < 0.01.

A retrospective analysis was conducted on two microarray datasets (GSE199841 and GSE81761) which included 71 PTSD samples and 43 control samples. PCA plot of the combined datasets “before batch correction” is illustrated in Figure 2A. Following the “after batch correction” using the “limma” R package, PCA plot demonstrated a uniform distribution (Figure 2B).

### 3.2. Landscape of the 60 RMGs in PTSD

Total 60 RMGs were extracted, including six m^6^A writers, fifteen m^6^A readers, two m^6^A erasers, eleven m^5^C writers, two m^5^C readers, four m^5^C erasers, two m^7^G writers, four m^1^A writers, one m^1^A eraser, and thirteen ψ regulators (Table S1). To explore the expression of RMGs in blood samples from individuals diagnosed with PTSD, an analysis of DEGs was initially conducted on the combined gene expression data matrix utilizing the limma package. The findings suggest differences in the expression levels of 60 RMGs between two groups (Figure 2C). Subsequently, 21 RMGs exhibiting statistically significant expression levels were identified through screening. Among them, five m^6^A regulators including RNA binding motif protein 15 (RBM15), Cbl proto-oncogene like 1 (CBLL1), YTH N6-methyladenosine RNA binding protein C1 (YTHDC1), YTH N6-methyladenosine RNA binding protein F3 (YTHDF3), and fragile X messenger ribonucleoprotein 1 (FMR1), five m^5^C regulators including NOP2/Sun RNA methyltransferase 4 (NSUN4), DNA methyltransferase 3 beta (DNMT3B), Tet methylcytosine dioxygenase 1/2/3 (TET1/2/3), one m^7^G regulators (WD repeat domain 4 (WDR4)), two m^1^A regulators including tRNA methyltransferase 10C (TRMT10C) and alpha-ketoglutarate dependent dioxygenase (ALKBH3), and three ψ regulators including pseudouridine synthase 7 (PUS7), pseudouridine synthase 7 like (PUS7L), and pseudouridine synthase 10 (PUS10) exhibited higher expression levels in PTSD compared to Con. In contrast, four m^6^A regulators including insulin like growth factor binding protein 1 (IGFBP1), insulin like growth factor binding protein 2 (IGFBP2), insulin like growth factor 2 mRNA binding protein 1 (IGF2BP1), and insulin like growth factor binding protein 1 (IGFBP1), alkB homolog 5 (ALKBH5), and one m^1^A regulators (tRNA methyltransferase 61A (TRMT61A)), exhibited higher expression levels in Con compared to PTSD. The majority of genes involved in RNA modifications exhibited a consistent expression profile, with genes responsible for RNA m^5^C modification regulators (NSUN4, DNMT3B, TET1, TET2, TET3) showing predominantly higher expression in individuals with PTSD compared to Con. A heatmap depicting the expression levels of the 21 identified genes was generated (Figure 2D).

CorrPlot correlations examined the interrelationships among RMGs. The analysis demonstrated robust positive correlations among the expression levels of readers, writers, erasers, and regulators. Specifically, PUS10 and PUS7, PUS10 and TRMT10C, and TRMT10C and ALKBH3 were found to be correlated genes, while TRMT61A displayed a negative correlation with the majority of other regulators (Figure 2E). The chromosomal locations of RMGs with significant differences are depicted in Figure 2F. The correlated correlation in the expression of RMGs indicates potential functional interactions, either synergistic or antagonistic, in the development of PTSD. Additionally, a protein–protein interaction network analysis was conducted on RMGs at http://string.embl.de/ (accessed on 7 July 2024). Proteins with an interaction score equal to or greater than 0.4 were selected for visualization (Figure 2G), providing additional evidence for the functional significance of RMGs in PTSD.

### 3.3. Construction of the Nomogram Models and RT-qRCR Validation

**Figure 3:**
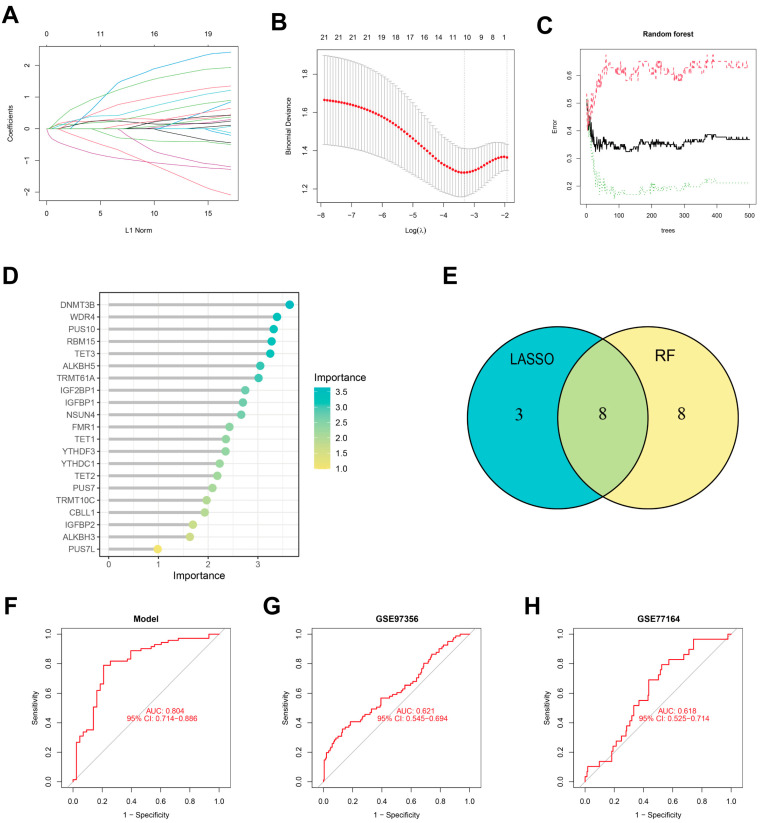
Identification of feature RMGs by Lasso and RF. (**A**) DEGs profiles based on LASSO coefficients. (**B**) LASSO coefficient values of the DEGs. The vertical dashed lines are the optimal log(λ) values. (**C**) The correlation plot between the number of RF trees and model error. (**D**) The Gini coefficient method in a random forest classifier yielded the following results. The importance index is on the *x*-axis, and the genetic variable is on the *y*-axis. (**E**) Venn plot displaying 8 overlapping feature RMGs selected by LASSO regression algorithm and random forest algorithm. (**F**–**H**) Receiver operating characteristic (ROC) curves for 8 feature RMGs in model (**F**), GSE97356 (**G**), and GSE77164 (**H**).

The LASSO (Figure 3A,B) and RF (Figure 3C,D) were used to identify feature RMGs among the twenty-one differential RMGs for the purpose of characterizing diseases and predicting PTSD incidence. LASSO regression analysis screened out 11 feature genes (Figure 3E); RF model analysis based on feature importance scores identified 16 feature genes with an importance score ≥ 2 as the threshold (Figure 3D,E). To obtain more robust RMGs, we intersected the feature genes selected by LASSO regression and RF, and finally identified eight feature genes: YTHDC1, IGFBP1, IGF2BP1, ALKBH5, NSUN4, TET2, TET3, and WDR4. The diagnostic model constructed based on these feature RMGs showed a high diagnostic performance (AUC = 0.804), as indicated by the ROC curve (Figure 3F). In addition, validation studies using two independent external validation sets further confirmed the good predictive accuracy of the scoring model (Figure 3G,H).

**Figure 4:**
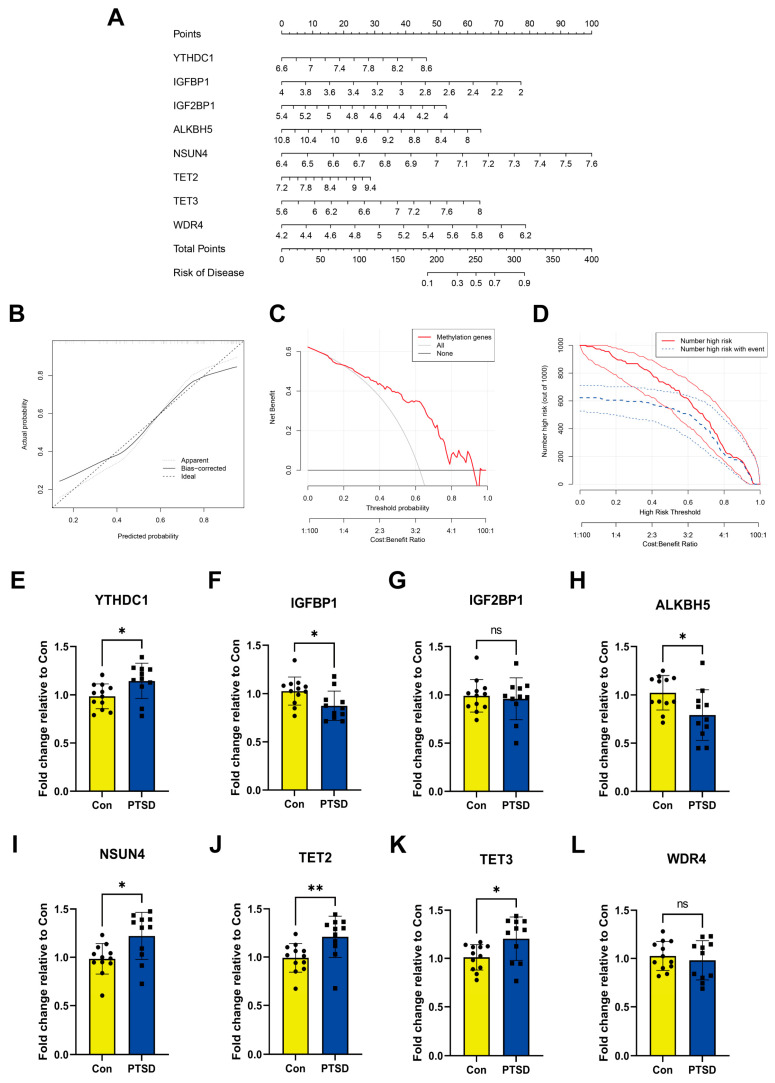
Construction of nomogram model and RT-qPCR experimental validation of feature RMGs. (**A**) Nomogram model. (**B**) Calibration curves. (**C**) Decision curve. (**D**) Clinical impact curve. (**E**–**L**) Relative mRNA expressions of 8 feature RMGs between Con and PTSD. Statistical annotations: ns, no significance; * *p* < 0.05; ** *p* < 0.01.

Then, we constructed a nomogram model of the feature RMGs for predicting the risk of PTSD (Figure 4A). Within the nomogram model, each gene is assigned an individual score, and the total score predicting PTSD incidence is calculated by summing these scores. Calibration curves showing strong agreement between predicted and observed out-comes (Figure 4B). The discrepancy between the red line representing the RMGs in the decision curve and the gray and black lines is apparent (Figure 4C). Furthermore, the clinical impact curve also demonstrated that the model provided strong predictive capability for prognosis (Figure 4D). Nevertheless, it is imperative to acknowledge the need for further validation utilizing more diverse and larger datasets to substantiate the prognostic precision of the results. To experimentally corroborate our bioinformatic findings, we performed RT-qPCR to assess the expression of the feature genes. YTHDC1, NSUN4 and TET2/3 in PTSD have higher expression compared to Con. Conversely, IGFBP1 and ALKBH5 showed lower expression in the PTSD compared to Con. There was no statistically significant difference in the expression level of other feature RMGs (Figure 4E–L). These findings were in alignment with part of bioinformatics analysis.

### 3.4. Two RMG Subtypes Identified by Differential RMGs

**Figure 5:**
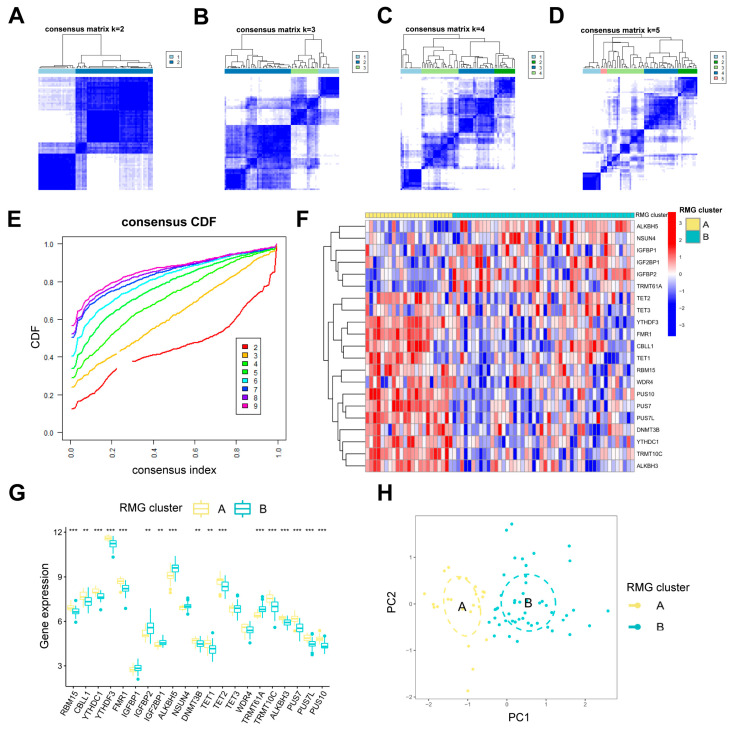
Clustering analysis of 21 differential RMGs related with PTSD. (**A**–**D**) The consensus matrices showing how the differential RMGs are grouped together for different k values between 2 and 5. (**E**) The k value of 2 shows the least decrease in the consensus CDF curve, suggesting the best clustering. (**F**,**G**) The expression heatmap and boxplot show the variation in expression of the differential RMGs between RMG cluster A and RMG cluster B. (**H**) PCA shows the unique RNA modification subtype expression patterns. Statistical annotations: ** *p* < 0.01, and *** *p* < 0.001.

Two distinct RNA modification patterns, RMG clusterA and RMG clusterB, were identified according to 21 differential RMGs through the utilization of the R package “ConsensusClusterPlus” (Figure 5A–E). RMG clusterA consisted of 23 cases, while RMG clusterB comprised 48 cases (Table S2). Subsequently, a heat map (Figure 5F) and histogram (Figure 5G) were generated to visually illustrate the variances in expression levels of the 21 differential RMGs between the two subtypes. It was noted that the expression levels of RBM15, CBLL1, YTHDC1, YTHDF3, FMR1, DNMT3B, TET1, TET2, TRMT10C, ALKBH3, PUS7, PUS7L, and PUS10 in RMG clusterA were higher compared to RMG clusterB. Conversely, the expression levels of IGFBP2, IGF2BP1, ALKBH5, and TRMT61A exhibited significantly higher differences in clusterB (Figure 5F,G). Additionally, the results of PCA indicated that the two RMG patterns could be differentiated based on differential RMGs (Figure 5H).

**Figure 6:**
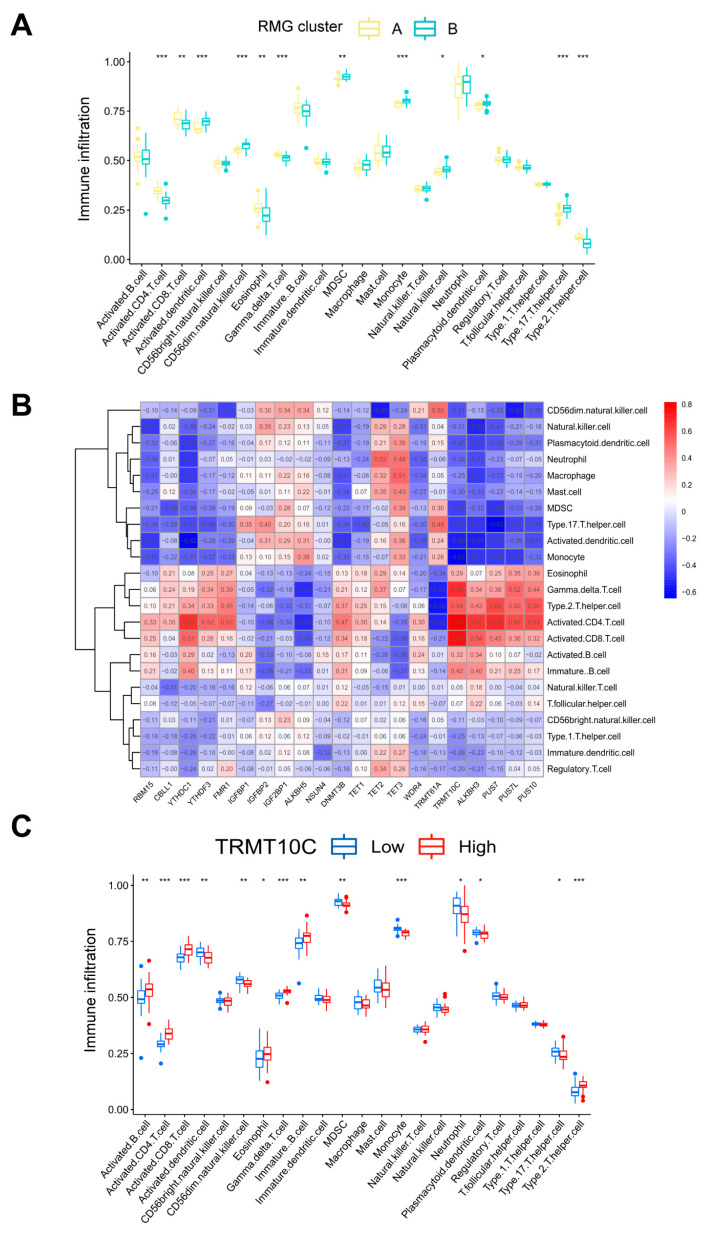
Analysis based on ssGSEA results. (**A**) Discrepancies in the infiltration of immune cells between RMG cluster A and RMG cluster B. (**B**) An immuno-correlation analysis revealing the relationship between immune cells and RMGs, with TRMT10C showing the strongest association with immune cells. (**C**) Differential immune cell infiltration between groups with lower and higher TRMT10C expression. Statistical annotations: * *p* < 0.05, ** *p* < 0.01, and *** *p* < 0.001.

Recent studies have revealed that abnormal immune inflammatory responses, such as elevated pro-inflammatory cytokines and dysregulation of immune cell function, constitute one of the core pathological mechanisms of PTSD [27,28]. Utilizing ssGSEA, we conducted an analysis of immune cell abundance in PTSD samples. Significant differences in infiltration levels of most immune cell subsets were observed between the two clusters, suggesting that RMG clusters A and B may represent distinct clinical subtypes of PTSD (Figure 6A). Cluster A demonstrated significantly higher levels of activated gamma delta T cell immunity (*p* < 0.001), activated CD8 T cells (*p* < 0.01), eosinophils (*p* < 0.01), activated CD4 T cells (*p* < 0.001), and Type 2 T helper cells (*p* < 0.001) in comparison to cluster B. This suggested that cluster A may exhibit a stronger inflammatory response and immune response. Furthermore, an evaluation of the correlation between 21 differential RMGs and immune cells was undertaken, as depicted in Figure 6B. The results indicated a strong association between TRMT10C and immune cells. A correlational analysis was performed to investigate the association between TRMT10C and immune cells, as depicted in Figure 6C. Specifically, there was a significant increase in immune cell infiltration of activated B cells (*p* < 0.01), activated CD4 T cells (*p* < 0.001), activated CD8 T cells (*p* < 0.001), eosinophil (*p* < 0.05), gamma delta T cell immunity (*p* < 0.001), immature B cell (*p* < 0.01), and Type 2 T helper cell (*p* < 0.001) in samples with higher TRMT10C expression.

**Figure 7:**
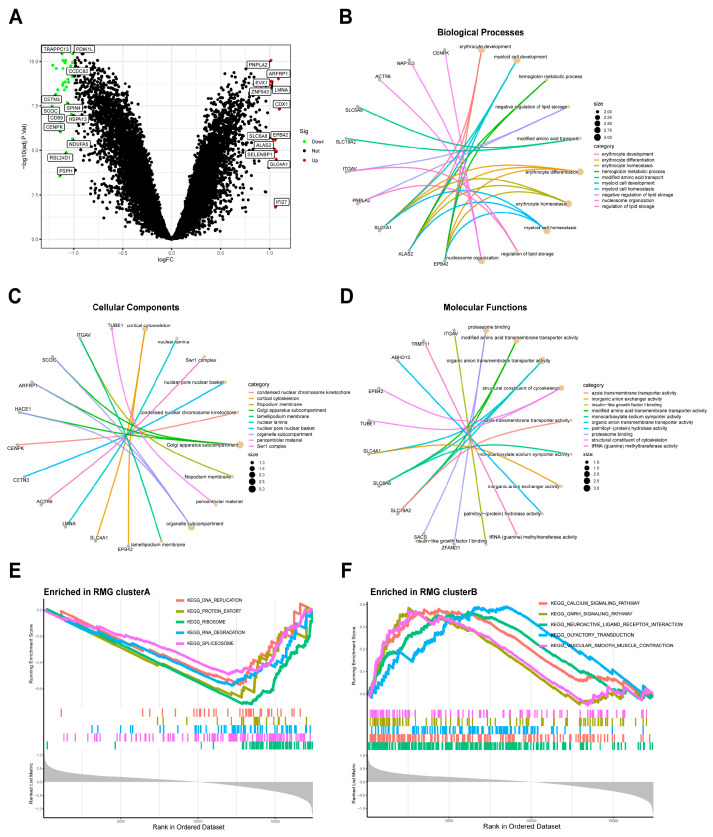
Identification and GO enrichment analysis of DEGs between two modification subtypes. (**A**) Volcano plot: The volcano plot was constructed using the logFC and Val. *p*-adjust. Red dots indicate upregulated genes; green dots indicate downregulated genes. (**B**) Top 10 enrichment terms in BP categories. (**C**) Top 10 enrichment terms in CC categories. (**D**) Top 10 enrichment terms in MF categories. (**E**,**F**) GSEA was conducted to analyze the enrichment levels of signaling pathways in the two clusters. The enrichment score line chart displays the running enrichment score on the vertical axis against the sorted genes on the horizontal axis. The highest point on the line chart represents the enrichment score of the gene group, with the gene preceding the peak being the central gene within the group. Genes within the group are indicated by lines in the center of the chart.

Using the specified criteria of |logFC| > 1 and adj. *p*. Val < 0.05, a comparative analysis was conducted between two RNA modification gene patterns in order to identify the intersection of DEGs. A total of 40 DEGs were identified between two patterns, as outlined in Table S3. Subsequently, we conducted GO enrichment analyses to elucidate the involvement of these DEGs. The differential gene expression levels were visualized using a volcano plot (Figure 7A), and the results of the GO enrichment analysis can be found in Table S4. A total of 148 GO terms were identified, with the top 10 biological processes (BPs), cellular components (CCs), and molecular functions (MFs) (Figure 7B–D). The research identified the metabolic and signaling pathways associated with differentially expressed genes. GO analysis determined that the BPs associated with the differentially expressed genes included erythrocyte development, myeloid cell development, erythrocyte differentiation, erythrocyte homeostasis, myeloid cell homeostasis, and nucleosome organization. The mainly enriched CCs included golgi apparatus subcompartment, organelle subcompartemnt, and cortical cytoskeleton. The MFs were mainly enriched organic anion transmembrane transporter activity, proteasome binding, modified amino acid transmembrane transporter activity, structural constitute of cytoskeleton. Additionally, the findings from the GSEA-KEGG database indicated significant enrichment of various pathways in the RMG clusterA compared to the RMG clusterB. These pathways included DNA replication, protein export, ribosome, RNA degradation, and spliceosome (Figure 7E). In contrast, the RMG clusterB exhibited enrichment in pathways such as calcium signaling, gnrh signaling, neuroactive ligand receptor interaction, olfactory transduction, and vascular smooth muscle contraction (Figure 7F). The exhaustive results of the GSEA-KEGG enrichment analysis are detailed in Table S5. The enrichment results of GO and GSEA-KEGG indicated that the identified differential genes are significantly associated with immune function and neurodevelopment.

### 3.5. Identification of Two RMG-Related DEG Subtypes and Consistency Check Between Two Genotyping Methods

**Figure 8:**
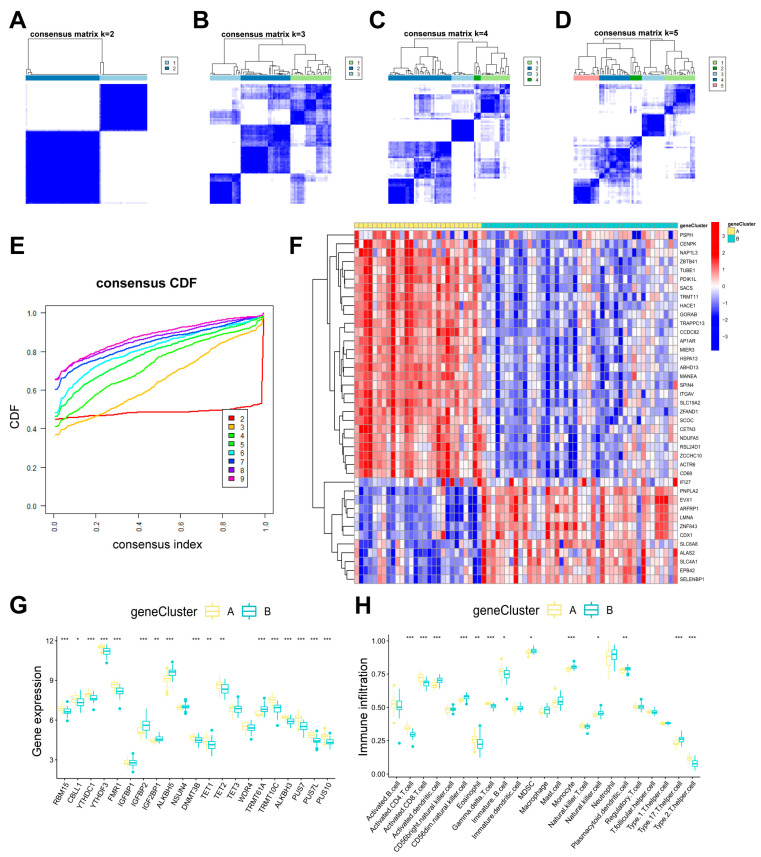
Consensus clustering of 40 RNA modification-related DEGs. (**A**–**D**) Consensus matrices of the 40 RNA modification-related DEGs for k values ranging from 2 to 5. (**E**) The k value of 2 had the smallest descending grade in the consensus CDF curve. (**F**) Gene clusterA and gene clusterB expression heat maps associated with 40 RNA modification-associated DEGs. (**G**) Boxplots showing the differential expression of 21 differential RMGs in gene clusterA and gene clusterB. (**H**) Distinct immune cell infiltration patterns observed in gene clusterA compared to gene clusterB. Statistical annotations: * *p* < 0.05, ** *p* < 0.01, and *** *p* < 0.001.

To analyze RNA modification patterns, a consensus clustering approach was employed to categorize PTSD cases into various genomic subtypes using 40 differentially expressed genes related to RMGs (Figure 8A–E). Two distinct RMG-related DEGs pat-terns were referred to as gene clusterA and gene clusterB (Table S6). The expression levels of the 40 RMG-related DEGs in two gene clusters were displayed in Figure 8F. The variance in expression levels of 21 differential RMGs and immune cell infiltration between two gene clusters closely mirrored the RMG patterns (Figure 8G,H). These findings further support the validity of our partitioning method using the consensus clustering approach.

**Figure 9:**
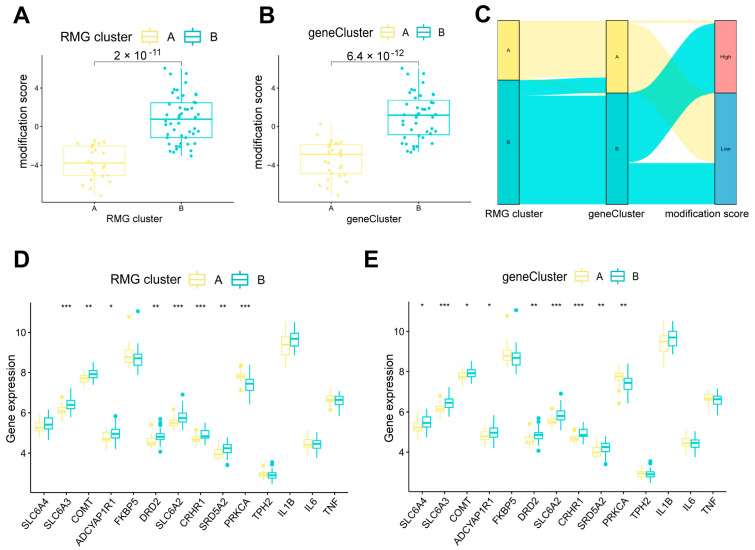
Role of RNA modification subtypes and its gene subtypes in distinguishing PTSD. (**A**) Differences in modification score between RMG clusterA and RMG clusterB. (**B**) Differences in modification score between gene clusterA and gene clusterB. (**C**) Sankey diagram showing the relationship between RNA modification subtypes, RNA modification gene subtypes, and RNA modification scores. (**D**) Differential expression levels of PTSD-related genes between RMG clusterA and RMG clusterB. (**E**) Differential expression levels of PTSD-related genes between gene clusterA and gene clusterB. Statistical annotations: * *p* < 0.05, ** *p* < 0.01, and *** *p* < 0.001.

The RNA modification scores for each sample within the two separate RMG clusters or gene clusters were computed using PCA algorithms to assess the RNA modification patterns (Table S7). Our analysis revealed that RMG clusterB or gene clusterB demonstrated a higher RNA modification score compared to RMG clusterA or gene clusterA (Figure 9A,B).

### 3.6. Role of RMG Patterns in Distinguishing PTSD

A Sankey diagram was utilized to visually represent the correlation between RNA modification scores, RMG patterns, and RMG-related DEGs patterns (Figure 9C). To investigate the relationship between RMG patterns and PTSD, we analyzed the association between RMG patterns and a set of specific genes hypothesized to play a role in PTSD [25]. It was noted that RMG clusterB or gene clusterB exhibited elevated levels of expression for SLC6A3, COMT, ADCYAP1R1, DRD2, SLC6A2, CRHR1, and SRD5A2 associated with nervous and endocrine systems compared to RMG clusterA or gene clusterA (Figure 9D,E), suggesting that cluster B exhibiting higher expression levels in multiple key genes highly relevant to the neurobiology of PTSD. Notably, these genes have been previously identified as being subject to RNA modification regulation in psychological disorders, with the exception of SRD5A2, as documented in existing literature [29,30].

Taken together, the distinct RNA modification patterns (RMG clusterA/B) and their corresponding gene expression subtypes (gene clusterA/B) delineate two putative subgroups of PTSD patients with divergent underlying pathophysiological mechanisms. ClusterA is characterized by an active immunoinflammatory phenotype, while clusterB exhibits a profile marked by elevated expression of neuroendocrine-related PTSD risk genes.

## 4. Discussion

In this study, we conducted a screening of 21 differentially expressed RMGs out of a total of 60 extracted RMGs from PTSD. These screened differentially expressed genes included nine m^6^A regulators, five m^5^C regulators, three m^1^A, one m^7^G regulators, and three ψ regulators. RBM15, CBLL1, YTHDC1, YTHDF3, FMR1, IGFBP1, NSUN4, DNMT3B, TET1, TET2, TET3, WDR4, TRMT10C, ALKBH3, PUS7, PUS7L, and PUS10 exhibited increased expression levels in individuals with PTSD, suggesting their potential roles in the biological mechanisms underlying the development and progression of the disorder. Conversely, IGFBP2, IGF2BP1, ALKBH5, and TRMT61A were downregulated in the PTSD. To further identify robust feature genes associated with RNA modifications, the feature genes selected by both LASSO regression and RF algorithms were intersected, yielding eight key feature genes: YTHDC1, IGFBP1, IGF2BP1, ALKBH5, NSUN4, TET2/3, and WDR4. The nomogram prognostic model constructed based on these eight feature genes demonstrated promising predictive performance. RT-qPCR further validated the expression of the aforementioned eight feature genes. The results revealed that the differential expression of genes including YTHDC1, IGFBP1, ALKBH5, NSUN4, and TET2/3 was in agreement with the bioinformatic analysis findings. In contrast, no significant expression differences were observed for the remaining two genes, which may be attributed to sample heterogeneity and limited sample size. Concurrently, genotyping of PTSD samples was performed using 21 differential RMGs and 40 DEGs associated with RMGs, respectively. Integrated assessment incorporating RMG expression profiles, immune cell abundance profiling, PCA, and PTSD-associated gene expression revealed high concordance between the two genotyping approaches. The results demonstrated that clusterB exhibited significantly elevated RNA modification scores, with previously reported PTSD-related genes showing marked upregulation in this cluster. These findings indicate that dysregulation of RNA modification machinery is critically involved in the pathogenesis of PTSD.

In recent years, the emergence of epigenome-wide association studies has enabled systematic associations between epigenetic variations and PTSD, providing mechanistic insights into the etiology of PTSD at the epigenetic level and identifying disease-relevant epigenetic loci. Leveraging this approach, multiple studies have not only uncovered DNA methylation signatures strongly linked to PTSD but also corroborated earlier findings in the field [31,32,33]. Moreover, although previous studies have identified numerous PTSD-associated biomarkers including susceptibility, diagnostic, and therapeutic biomarkers [4], their clinical applicability remains limited due to substantial phenotypic heterogeneity among individuals with PTSD and frequent psychiatric comorbidities [34]. These factors contribute to inconsistent biomarker performance and hinder their utility in clinical practice for diagnosis, treatment guidance, or prognosis prediction [35]. Notably, while existing research has extensively explored epigenetic mechanisms in PTSD, such as DNA methylation and noncoding RNAs [32,36,37,38,39,40,41,42,43,44,45], the role of RNA modifications, as a crucial layer of epigenetic regulation, has remained largely uninvestigated. In light of these gaps, our study provides the first evidence that several RNA modifications including m^6^A, m^5^C, m^1^A, m^7^G, and ψ are significantly associated with PTSD. This breakthrough not only offers novel insights into the molecular underpinnings of PTSD but also highlights the potential of RNA modifications as clinically translational biomarkers for the disorder.

Given the accessibility and simplicity of experimental design, phenotypic research on PTSD has predominantly relied on animal models, with the conditioned fear paradigm being the most classical among them [46]. This model allows for precise control over trauma intensity and timing, effectively recapitulating the persistence and generalization of fear memory [47], which closely mirrors the key symptomatology of PTSD. Through its application, epigenetic mechanisms associated with PTSD have been progressively elucidated. Notably, this animal model has unveiled a range of PTSD-associated RNA modifications, including RNA m^6^A and m^5^C methylation. In one study utilizing long-read Oxford Nanopore Technologies direct RNA sequencing, researchers constructed a comprehensive RNA modification map of the amygdala in a PTSD mouse model [22]. The analysis revealed significant enrichment of RNA modifications in genes related to PTSD pathogenesis, with the majority of identified modifications following established patterns associated with m^6^A methylation [22], which plays an important role in brain development, physiology, and pathological processes [48]. In our present study, four m^6^A feature regulators (YTHDC1, IGFBP1, IGF2BP1, and ALKBH5), critically implicated in psychiatric disorders and neurodegenerative diseases [49,50,51], were identified to be associated with PTSD. The four regulatory factors identified herein are implicated in distinct aspects of mRNA metabolism, including splicing, nuclear export, translation, and degradation [52,53]. Moreover, behavioral studies in animal PTSD-like models have demonstrated that specific m^6^A regulators, such as methyltransferase-like 3, Methyltransferase-like 14, and Wilms’ tumor 1-associating protein, are involved in fear conditioning [23,24]. Integrating our results with previous findings, we propose that the four m6A regulatory factors identified in this study may contribute to epigenetic mechanisms associated with PTSD.

Furthermore, our study suggests that RNA m^5^C methylation may serve as a potential regulatory layer in the pathogenesis of PTSD. We observed altered expression of key m^5^C writers (NSUN4) and erasers (TET family enzymes: TET2, and TET3) in PTSD, suggesting a dynamic and dysregulated RNA modification landscape in response to traumatic stress. These findings align with recent studies linking m^5^C to stress vulnerability and cognitive processes [54,55], yet they also underscore the complexity of epitranscriptomic regulation. Specifically, neuronal deficiency in NSUN2 has been shown to alter tRNA methylation landscapes, leading to proteomic shifts that disrupt synaptic signaling and PTSD-like behaviors [55]. Similarly, TET2, in complex with upstream frameshift 1, has been demonstrated to modulate stress-sensitive mRNA stability, and its elevated expression could contribute to aberrant stress response gene regulation under traumatic conditions [54]. This observation intriguingly parallels findings from PTSD patients involving the related enzymes TET2. More interestingly, under chronic mild stress conditions, only TET2 exhibited increased mRNA and protein levels among the TET family members, whereas both TET1 and TET3 showed reductions [54]. In addition, deficiency of TET3 exacerbates fear generalization in mice [56], a pattern sharply contrasting with our bioinformatic predictions. This discrepancy may be attributed to interspecies differences and the inherent limitations of animal stress models in fully recapitulating the complex etiology of PTSD.

Our current study reveals elevated WDR4 mRNA expression in patients with PTSD, suggesting a potential role in the molecular pathology of this disorder. WDR4, a functional methyltransferase in m^7^G methylation complex with METTL1, is frequently associated with neurodevelopmental disorders, including microcephalic primordial dwarfism [57,58] and Down syndrome [59,60]. Previous findings suggest that WDR4-mediated tRNA m^7^G plays a crucial role in regulating neuroectoderm commitment in embryonic development, as well as in preserving cognitive function and hippocampal plasticity [61]. Our observation of its upregulation in PTSD and the functions of WDR4 mentioned above may imply that this epitranscriptomic mechanism may also contribute to maladaptive neural plasticity and stress response dysregulation characteristic of PTSD. However, the precise functions and mechanisms of WDR4 in PTSD remain to be further explored and validated experimentally.

Our study delineated two distinct molecular subtypes of PTSD including clusterA and clusterB, primarily characterized by divergent immune infiltration patterns and differential expression of previously reported PTSD-associated genes. Previous studies indicated that the function and distribution of immune cells may be altered in PTSD [62,63]. Our study showed that clusterA displays a more robust and hyperactive immunoinflammatory profile, which represents a subtype characterized by elevated immune infiltration and enhanced activation status and may secret relevant pro-inflammatory cytokines consistent with previous findings [64]. Of interest is the fact that individuals with PTSD exhibited significantly elevated concentrations of classic pro-inflammatory biomarkers compared to non-PTSD controls, notably including IL1β, IL6, IL17, TNF-α, interferon-γ, and C-reactive protein [41,65]. Moreover, peripheral levels of these pro-inflammatory markers demonstrated positive correlations with both the severity of PTSD symptomatology and an increased likelihood of PTSD development [66,67]. Therefore, immune inflammation is not simply an epiphenomenal correlate of PTSD, but rather may constitute a pivotal pathological mechanism that drives both disease progression and clinical severity. In parallel, anti-inflammatory medications, including non-steroidal anti-inflammatory drugs, cytokine-targeting monoclonal antibodies, and angiotensin system modulators, have been clinically employed to alleviate symptoms in patients with PTSD by attenuating inflammatory responses [68]. However, a subset of patients with PTSD exhibits significant elevations in systemic inflammatory markers, while another subset demonstrates normal or even reduced levels of inflammation [69]. This discrepancy suggests the potential existence of inflammation-related heterogeneity in PTSD.

Correspondingly, clusterB demonstrates a fundamentally different profile. It might represent significantly higher expression of canonical PTSD-risk genes involved in neurotransmitter signaling, stress hormone response, and neuroendocrine function. This pattern aligns more closely with traditional models of PTSD that emphasize dysregulation of the hypothalamic–pituitary–adrenal axis and monoaminergic systems [25]. It should be noted that these highly expressed genes in clusterB demonstrate significant associations with the risk of developing PTSD or symptom severity [70,71,72,73,74,75,76]. Importantly, gene-environment interactions and epigenetic regulation also play critical roles in this pathogenic process. Based on these findings, this dichotomy between clusterA and clusterB provides a novel molecular framework for understanding PTSD heterogeneity. It is plausible that patients in clusterA may present with more prominent immunoinflammation or could potentially benefit from immunomodulatory therapies. Conversely, clusterB patients might exhibit symptoms more closely tied to neuroendocrine dysregulation and may respond differently to existing pharmacological interventions targeting neurotransmitter systems. Future studies correlating these molecular subtypes with detailed clinical symptom clusters, trauma histories, and treatment outcomes are essential to validate their clinical utility.

To our knowledge, this represents the first study to focus on establishing the classification and diagnostic value of RNA modifications in PTSD through transcriptome-wide mapping, including m^5^C, m^6^A, m^1^A, m^7^G, and ψ. While this study provides evidence supporting the association between RMGs, immune infiltration, and PTSD, several limitations should be acknowledged. First, the observed correlations do not imply causality, and the mechanistic links among these factors remain speculative. Future studies employing functional experiments, such as in vitro or in vivo knockdown/overexpression models, are necessary to establish causal relationships and elucidate underlying molecular pathways. Second, the moderate sample size may limit the statistical power to detect subtle associations or subgroup effects. Additionally, the use of peripheral blood samples, rather than brain tissues, restricts the direct interpretation of findings in the context of psychiatric illness processes, although peripheral biomarkers remain relevant for their translational potential, owing to the fact that all cells in the human body share an identical genomic sequence [77]. To address these limitations, future investigations will prioritize cross-tissue validation and large-scale multi-center cohorts. Finally, the cross-sectional design of our study precludes the assessment of temporal dynamics or disease progression-related changes in RMG and immune profiles. Longitudinal studies with repeated measurements will be valuable to capture the evolving nature of PTSD-associated epitranscriptomic and immune alterations.

## 5. Conclusions

Through the integration of machine learning and bioinformatic analyses, we identified 21 differentially expressed RNA modification regulators. From these, eight signature RMGs were selected LASSO regression combined with RF algorithms to construct a predictive model for PTSD risk. Subsequent investigation of RNA modification patterns using the 21 RMGs suggested two possible subtypes, characterized by high immunoinflammation and high-neuroendocrine dysregulation. Furthermore, our findings provide novel therapeutic implications for immunomodulatory interventions and neurotransmitter-targeted pharmacotherapy in PTSD. Collectively, these results underscore the translational promise of RNA epitranscriptomic mechanisms in both PTSD pathogenesis, immune treatment development and neurotransmitter system interventions.

## Figures and Tables

**Table 1:** Detail information of two training microarray datasets.

GEO Accession	Sequencing Type	Healthy Controls	PTSD	Source Tissue
GSE199841	Array	16	32	Venous blood
GSE81761	Array	27	39	Venous blood

**Table 2:** Detailed clinical information comparing PTSD and control group.

Group	Sample Size	Gender (Male/Female)	Age (Median (Interquartile Range))
PTSD	11	4/7	25 (20–32)
Control	12	4/8	25.5 (23–31)

**Table 3:** Sequences of RMGs primers used for RT-qPCR.

Genes (Homo Sapiens)	Forward Primer (5′-3′)	Reverse Primer (5′-3′)
GAPDH	GTCTCCTCTGACTTCAACAGCG	ACCACCCTGTTGCTGTAGCCAA
YTHDC1	AACTGGTTTCTAAGCCACTGAGC	GGAGGCACTACTTGATAGACGA
IGFBP1	TTGGGACGCCATCAGTACCTA	TTGGCTAAACTCTCTACGACTCT
IGF2BP1	CAAAGGAGCCGGAAAATTCAAAT	CGTCTCACTCTCGGTGTTCA
ALKBH5	CGGCGAAGGCTACACTTACG	CCACCAGCTTTTGGATCACCA
NSUN4	CCATCAATCCGTGTCAGTCTC	GCTTAGCACTTACATGATCCCAG
TET2	GATAGAACCAACCATGTTGAGGG	TGGAGCTTTGTAGCCAGAGGT
TET3	GCCGGTCAATGGTGCTAGAG	CGGTTGAAGGTTTCATAGAGCC
WDR4	CCACCTCCATAGCAAGCAGTG	ACGCTTACTGTCATCGGTTAAAG

## Data Availability

The datasets utilized in this study can be accessed from the GEO datasets repository at https://www.ncbi.nlm.nih.gov/geo/ (accessed on 5 June 2024).

## Supplementary Materials

The following supporting information can be downloaded at: https://www.mdpi.com/article/10.3390/diseases13100323/s1, Table S1: Sixty RNA modification regulators genes were collected in this study. Table S2: Two distinct RNA modification patterns. Table S3: 40 DEGs were identified between two patterns. Table S4: GO enrichment analysis. Table S5: Exhaustive results of the GSEA-KEGG enrichment analysis. Table S6: Two distinct RNA modification geneCluster. Table S7: RNA modification scores for each sample within the two separate RMG clusters.

## Abbreviations

The following abbreviations are used in this manuscript:

PTSD	Post-traumatic disorder
m^6^A	N6-methyladenosine
m^5^C	5-methylcytosine
m^1^A	N1-methyladenosine
m^7^G	N7-methylguanosine
ψ	Pseudouridine
RMGs	RNA modification-related genes
PUS10	Pseudouridine Synthase 10
TET1	Tet methylcytosine dioxygenase 1
WDR4	WD repeat domain 4
YTHDF3	YTH N6-methyladenosine RNA binding protein F3
TRMT10C	tRNA methyltransferase 10C
IGFBP1	Insulin like growth factor binding protein 1
ALKBH5	AlkB homolog 5
FMR1	Fragile X messenger ribonucleoprotein 1
TET3	Tet methylcytosine dioxygenase 3
RBM15	RNA binding motif protein 15
TET2	Tet methylcytosine dioxygenase 2
CBLL1	Cbl proto-oncogene like 1
DNMT3B	DNA methyltransferase 3 beta
TRMT61A	tRNA methyltransferase 61A
IGFBP2	Insulin like growth factor binding protein 2
NSUN4	NOP2/Sun RNA methyltransferase 4
PUS7L	Pseudouridine synthase 7 like
YTHDC1	YTH N6-methyladenosine RNA binding protein C1
PUS7	Pseudouridine synthase 7
ALKBH3	AlkB homolog 3, alpha-ketoglutarate dependent dioxygenase
IGF2BP1	Insulin like growth factor 2 mRNA binding protein 1
MDSCs	Myeloid-derived suppressor cells
GEO	Gene Expression Omnibus
GO	Gene Ontology
DEGs	Differentially expressed genes
ssGSEA	Single sample gene set enrichment analysis
RT-qPCR	Real-time quantitative polymerase chain reaction
RMA	Robust multiarray analysis
PCA	Principal component analysis
RF	Random forests
LASSO	Least absolute shrinkage and selection operator
ROC	Receiver operating characteristics
DCA	Decision curve analysis
CDF	Consensus cumulative distribution function
GSEA-KEGG	Gene set enrichment analysis and Kyoto encyclopedia of genes and genomes
SLC6A4	Solute carrier family 6 member 4
SLC6A3	Solute carrier family 6 member 3
COMT	Catechol-O-methyltransferase
ADCYAP1R1	ADCYAP receptor type I
FKBP5	FKBP prolyl isomerase 5
DRD2	Dopamine receptor D2
SLC6A2	Solute carrier family 6 member 2
CRHR1	Corticotropin releasing hormone receptor 1
SRD5A2	Steroid 5 alpha-reductase 2
PRKCA	Protein kinase C alpha
TPH2	Tryptophan hydroxylase 2
IL1β	Interleukin 1 beta
IL6	Interleukin 6
TNF	Tumor necrosis factor
HPBMs	Human peripheral blood monocytes
BP	Biological process
CC	Cell component
MF	Molecular function
TET	Ten-eleven translocation
